# A case of eosinophilic dermatosis of hematologic malignancy treated with omalizumab

**DOI:** 10.1016/j.jdcr.2026.03.049

**Published:** 2026-04-02

**Authors:** Raveena Ghanshani, Grace Chan, Brittney DeClerck, Gene H. Kim, Ashley B. Crew, Katrina Lee, Jennifer L. Hsiao

**Affiliations:** a Keck School of Medicine of USC, University of Southern California, Los Angeles, California; b Department of Dermatology, University of Southern California, Los Angeles, California

**Keywords:** chronic lymphocytic leukemia, eosinophilic dermatoses, eosinophilic dermatosis of hematologic malignancy, insect bite-like reaction, omalizumab

## Introduction

Eosinophilic dermatosis of hematologic malignancy (EDHM) is a rare pruritic skin eruption linked to several hematologic malignancies, such as chronic lymphocytic leukemia, large cell lymphoma, and acute lymphoblastic leukemia. EDHM is most commonly seen in patients with chronic lymphocytic leukemia, constituting up to 77% of EDHM cases.^1^ EDHM may present with varying skin lesions, including papules, bullae, plaques, and nodules, typically on the extremities but can occur anywhere on the body. Eosinophilic infiltrate is characteristically seen on histopathology. There is a paucity of data regarding treatment options for EDHM, and the efficacy of therapies in existing literature is mixed, marked by frequent recurrence of lesions.^2^ Literature is limited mostly to case reports and series, which describe the use of treatments such as topical and systemic corticosteroids, antihistamines, doxycycline, nicotinamide, phototherapy, dupilumab, and omalizumab, for the treatment of EDHM.^2^, ^3^, ^4^, ^5^, ^6^ Herein, we report a refractory case of EDHM that responded to omalizumab.

## Case report

**Fig 1:**
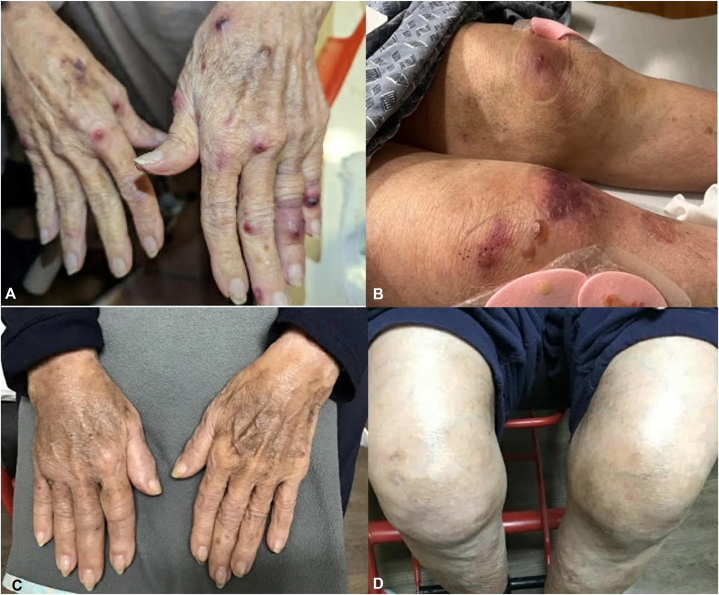
Tense bullae and edematous erythematous papules scattered on extremities before (**A, B**) and 15 months after (**C, D**) treatment with omalizumab.

**Fig 2:**
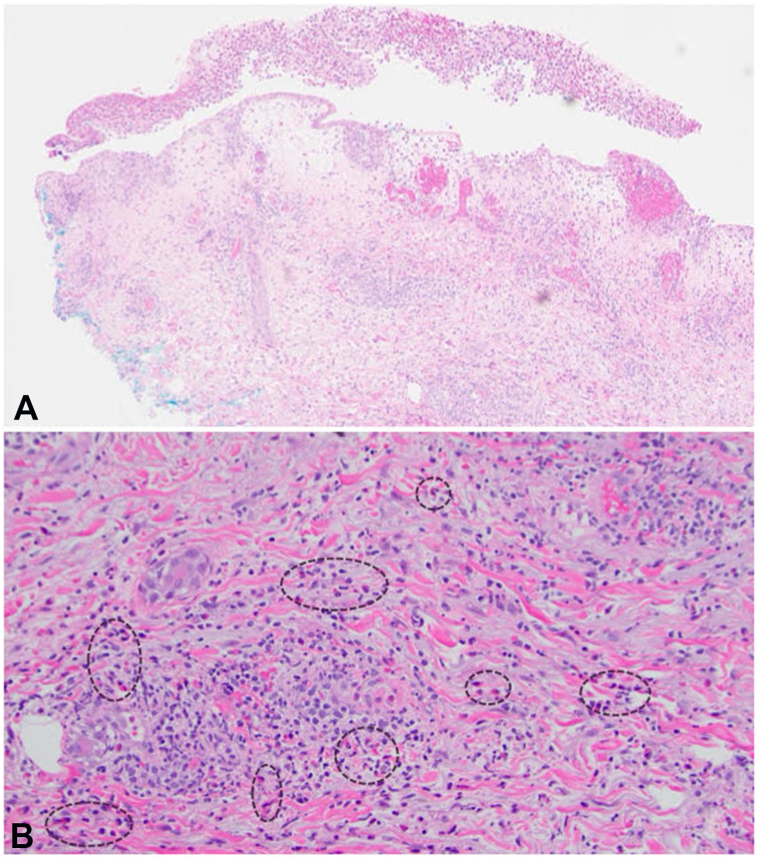
Punch biopsy results showing superficial and deep perivascular and interstitial inflammation with numerous eosinophils at **(A)** 40× and **(B)** 400× magnification.

An 89-year-old female presented to dermatology clinic with a 1-year history of intermittent pruritic blisters predominantly on the extremities. The patient had been hospitalized several times with concern for recurrent cellulitis. The patient’s medical history was notable for chronic lymphocytic leukemia (CLL), for which she was taking acalabrutinib. For her pruritus, she failed to improve with loratadine, cetirizine, hydroxyzine, famotidine, and empiric scabies treatment. Physical examination revealed tense bullae and edematous erythematous papules on her extremities (Fig 1). Differential diagnosis included bullous pemphigoid, neutrophilic eccrine hidradenitis, bullous arthropod reaction, and drug-induced bullous dermatosis such as bullous sweet syndrome. Lab work included WBC (4.76), RBC (3.10), Hgb (9.7), platelet count (90), and absolute eosinophil count (310 cells/mcL). Punch biopsies showed subepidermal vesiculation, epidermal spongiosis and erosion, and superficial and deep perivascular and interstitial inflammation with numerous eosinophils (Fig 2). Direct immunofluorescence and bullous pemphigoid antigens 1 and 2 were negative (tests were performed when patient was not on systemic steroids). Notably, the patient’s serum IgE was highly elevated at 11,905 IU/mL. The patient was diagnosed with eosinophilic dermatosis of hematologic malignancy.

The patient was started on an oral prednisone taper starting at 60 mg daily and had a good response with improvement of her skin lesions but continued to have intermittent flares. She was started on dupilumab 300 mg every 2 weeks as well as narrow band ultraviolet B (NB-UVB) phototherapy 3 times per week in an effort to transition to a steroid-sparing treatment regimen. Despite 3 months of this therapy, her lesions would recur whenever the steroid was tapered off. During this time, the patient was also switched from acalabrutinib to pirtobrutinib and noted to have stable CLL. Both dupilumab and NB-UVB were then discontinued, and the patient was started on omalizumab 300 mg every 4 weeks while on a new prednisone 60 mg daily taper. The patient tolerated omalizumab well with no side effects and the prednisone was able to be tapered off. Complete resolution of her skin findings was noted within 7 to 8 months of starting omalizumab as well as significant improvement in her pruritus. Given the patient still had some itch on the 300 mg every 4 weeks omalizumab dose, her dose frequency was increased to 600 mg every 4 weeks and then ultimately to 600 mg every 2 weeks with good control of symptoms and no adverse events. More than a year and a half after the first injection, the patient’s disease remains overall well-controlled on omalizumab.

## Discussion

Cases of chronically relapsing-remitting lesions that mimic arthropod bites in patients with underlying hematologic malignancy such as CLL should prompt consideration for the diagnosis of EDHM. In some cases, this skin condition may be the sign of an undiagnosed hematologic malignancy.^7^ This condition, first detailed by Weed in 1965, was historically thought to be exaggerated hypersensitivity reactions to arthropod bite lesions.^8^ The role that insect bites may play in the pathogenesis of EDHM remains unclear.^7^

Current treatment options for EDHM are limited and no standardized guidelines exist. One systematic review found that systemic and topical corticosteroids as well as treatment of the underlying malignancy were the most frequently utilized strategies for EDHM.^2^ The clinical course of EDHM is variable, with some patients demonstrating improvement with chemotherapy, while others do not. In 1 retrospective study of 37 patients, mean duration between hematologic and EDHM diagnosis was 57 months, mean duration of EDHM rash was found to be 7 months (range 1-34 months), and mean relapse-free interval was 5 months.^9^ In many case reports, lesions improved with oral steroids but would then recur after tapering. Although steroids have been a mainstay of short-term treatment for EDHM, biologics are an emerging therapeutic approach that should be considered in patients who may need a long-term therapeutic option.

Although not fully understood, it is speculated that EDHM results from immune dysfunction secondary to the underlying malignancy, leading to increased Th2 inflammation with higher levels of IL-4 and IL-5.^10^ These cytokines play a role in eosinophil recruitment, predisposing patients to eosinophilic dermatoses. Thus, medications such as dupilumab and omalizumab which help target Th2 inflammation may reduce the manifestations of disease. Omalizumab, a recombinant humanized IgG1 monoclonal antibody to IgE, has also been used off-label for the treatment of IgE-mediated dermatologic conditions such as atopic dermatitis. Further research is needed to determine whether IgE levels in patients with EDHM may predict omalizumab efficacy or guide treatment selection.

Our case suggests that omalizumab may be a promising and safe long-term treatment modality for patients with EDHM. To our knowledge, only 1 other case report in the literature describes the utilization of omalizumab as maintenance therapy for EDHM in a 62-year- old patient with B-cell CLL with EDHM whose pruritus improved on narrow-band UVB phototherapy twice a week and omalizumab 300 mg injections monthly.^5^ Our patient’s disease has overall remained well-controlled with 600 mg every 2 weeks dosing.

EDHM is a rare dermatosis with a paucity of data on treatment efficacy. Improved understanding of the pathophysiology of this condition will help guide treatment selection. Omalizumab may be considered as a treatment option in patients with recalcitrant, chronic disease.

## Conflicts of interest

Crew has served as an investigator for AstraZeneca and Incyte. Lee has served as an investigator for Novartis. Hsiao is on the board of directors for the HS Foundation and has served as an advisor, investigator, and/or speaker for AbbVie, Aclaris, AstraZeneca, Boehringer Ingelheim, Galderma, Incyte, Navigator Medicines, Novartis, Pfizer, Regeneron, Sanofi, UCB. Ghanshani, Chan, DeClerck, and Kim report no disclosures.
